# Informal Rural Eldercare Solutions: The Emergence and Implications of Family-Care Homes in Shanghai’s Periphery

**DOI:** 10.1093/geroni/igaf122.2832

**Published:** 2025-12-31

**Authors:** Ziqi Zhang, Linzhi Su

**Affiliations:** Shanghai Jiao Tong University, Shanghai, Shanghai, China (People’s Republic); Shanghai Jiao Tong University, Shanghai, Shanghai, China (People’s Republic)

## Abstract

This study faces the structural tension between declining family-based care systems and inadequate formal care provisions in rural China by centering on self-organized family-care homes in Shanghai’s peri-urban villages. We employ mixed methods—snowball sampling of 14 cases, 70 hours of environmental-behavioral mapping, and semi-structured interviews with 16 operators and 10 older residents—to analyze how these informal facilities navigate spatial production and regulatory constraints. Through integrated graphic analysis, thematic coding, and fsQCA, three critical dimensions were focused: (1) Spatial hybridization achieves cost-efficiency via residential retrofitting (flexible partitions, ambiguous but adaptive cohabitation units) that blend dwelling, caregiving, and livelihood functions; (2) Relational reconfiguration constructs place-based care networks through quasi-familial ties (operator-resident-local staff triads) and context-sensitive service portfolios integrating daily support, emotional bonding, and intergenerational exchange; (3) Institutional negotiation leverages policy ambiguities (exploiting ambiguities in homestead policies) and tacit trust mechanisms to bypass rigid regulatory frameworks, fostering grassroots innovation within governance gaps. The study proposes integrating such practices into an extended mutual-aid care framework for legitimacy and advocates for a community-embedded micro-institution certification system with environmental design guidelines in rural areas. It expands the theoretical understanding of “quasi-formal care spaces” in gerontological geography, as well as enriches institutional and environmental design innovation scholarship on rural aging-in-place in developing contexts.

